# Planning for assisted colonization of plants in a warming world

**DOI:** 10.1038/srep28542

**Published:** 2016-06-27

**Authors:** Alessandro Ferrarini, Alberto Selvaggi, Thomas Abeli, Juha M. Alatalo, Simone Orsenigo, Rodolfo Gentili, Graziano Rossi

**Affiliations:** 1 Department of Earth and Environmental Sciences, University of Pavia, via S. Epifanio 14, I-27100 Pavia, Italy; 2 I.P.L.A., Corso Casale 476, I-10132 Torino, Italy; 3 Department of Biological and Environmental Sciences, College of Arts and Sciences, Qatar University, P.O. Box 2713, Doha, Qatar; 4 Department of Agricultural and Environmental Sciences - Production, Landscape, Agroenergy, University of Milan, Via Celoria 2, I-20122 Milano, Italy; 5 Department of Environmental and Territory Science, University of Milano-Bicocca, Piazza della Scienza 1, I-20126 Milano, Italy

## Abstract

Assisted colonization is one way of facilitating range shifts for species that are restricted in their ability to move in response to climate change. Here we conceptualize and apply a new decision framework for modelling assisted colonization of plant species prior to *in situ* realization. Three questions were examined: a) Is species translocation useful in a certain area? b) where, and c) how long will it be successful in the future? Applying our framework to *Carex foetida* in Italy at the core of its distribution and its southern edge revealed that assisted colonization could be successful in short-term (2010–2039) climate conditions, partially in medium (2040–2069) but not in long-term (2070–2099) scenarios. We show that, for some species, it is likely that assisted colonization would be successful in some portions of the recipient site under current and short-term climate conditions, but over the mid- and long-term, climate changes will make species translocation unsuccessful. The proposed decision framework can help identify species that will need different conservation actions (seed banks and/or botanical gardens) when assisted colonization is unlikely to be successful. Furthermore it has broad applicability, as it can support planning of assisted migration in mountainous areas in the face of climate change.

Assisted colonization involves translocating species populations outside their current distribution when their ability to survive in their natural habitat in the face of future climate change is threatened^1^. There are three alternative scientific views on species translocation^2^: i) aggressive assisted colonization is needed and should include extensive translocation of species also beyond their current distributions; ii) assisted colonization should be avoided because of the difficulties in predicting target regions for assisted colonization, the lack of available data for modelling the climate envelopes of most species, and uncertainties in climate predictions; iii) constrained assisted colonization should be carried out by balancing the benefits and risks associated with assisted colonization. Many authors support constrained assisted colonization, claiming that it will be an essential tool for species conservation in a changing climate^3^,^4^. This latter approach to assisted colonization requires a modelling framework to produce effective management plans for species translocation. Following this position, some authors have pointed out that biogeography may help solve many problems concerning risk assessment of species translocation^5^. Translocation of species outside their current distribution, but within the same biogeographical range where they evolved, would maintain the presence of their evolutionary drivers^6^.

However, even the biogeographical approach does not solve one of the major weaknesses of assisted colonization, namely potential medium and long-term failure of translocation^7^.

Accordingly, in this study we conceptualized and applied a new framework for spatio-temporal modelling of the assisted colonization of plant species prior to *in situ* realization. Three specific questions were studied: a) how much area of the recipient site is suitable for translocation of the target species? b) in which areas of the recipient site will assisted colonization be most profitable in the future? and c) how long will the assisted colonization be successful under climate change scenarios? We applied our framework to the target species *Carex foetida* All., an alpine species of conservation interest in Italy due to its vulnerability to global warming, that reaches its southern distribution in the N-Apennines (a mountain range characterized by low altitude summits). We sought to determine the spatio-temporal feasibility of assisted colonization of this species in climatic conditions near to its southernmost distribution boundary.

## Results

### Core area

In the Western Alps (Piemonte region), 227 growing sites of *C. foetida* were identified during field surveys (Fig. S1). After minimizing the spatial autocorrelation, 202 sampling points remained.

Three climate variables (AHM, DD0 and MAT; Table 1) had little predictive power (permutation test scores <1) and hence we filtered these out. The optimized set of five remaining climate variables (FFP, MAP, PAS, SHM, TAVE_SM; Table 1) was then used to define the climate profile of the study species in the core area. The mean climate vector for *C. foetida* in the core area resulted: <107.9, 1206.0, 517.5, 20.77, 9.04> (Table S1). SHM was the most important predictor (permutation test score = 54%), followed by TAVE_SM and MAP with similar scores. The response curves (Fig. S2) showed that site suitability for *C. foetida* generally increases with MAP, while the other variables provided a contribution increasing up to a peak, followed by a rapid decrease. The overall AUC score for train data was 0.961, while it resulted 0.960 for test data (Fig. S2). These high AUC values indicate that the distribution of *C. foetida* was well explained by the optimized set of climate variables employed here.

### Peripheral area

**Figure 1:**
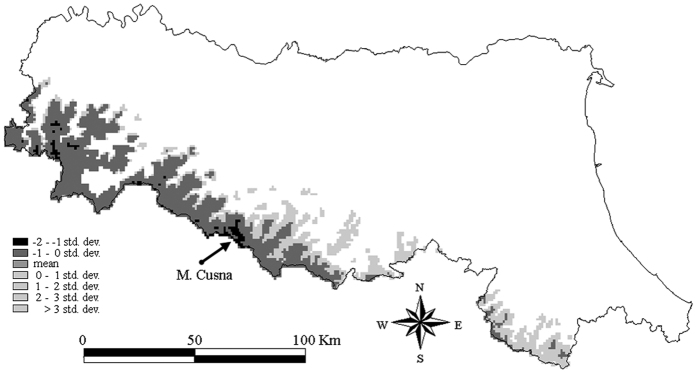
Climate similarity (measured as Mahalanobis distance) of the mountain system (areas >800 m a.s.l.; 3520 km^2^) of the Emilia-Romagna region with respect to the mean vector of the optimized set of climate variables used to profile *Carex foetida* in the core area. Distances are expressed as standard deviation from the average distance. Negative values indicate higher similarity with respect to the climate conditions of *C. foetida* in the core area, positive values higher dissimilarity. White pixels correspond to non-mountain areas. Map was created using GRASS GIS^25^.

The area around M. Cusna (2120 m a.s.l.; Fig. S3) resulted the portion of the N-Apennines (Emilia-Romagna region) with highest similarity with respect to the climate profile of *C. foetida* in the core area (Fig. 1). This area extends over 575 ha (about 2500 m × 2300 m; barycentre coordinates: 44°15′N, 10°24′E), at altitude 1370–2120 m a.s.l. It resulted in a Mahalanobis distance from the climate profile of *C. foetida* in the core area of 4.69 ± 0.15 (*n *= 550 pixels; Fig. S4), which is about 1.5 standard deviations lower than the average distance (10.38 ± 3.81, *n *= 352,000 pixels) in the mountain system of Emilia-Romagna (Fig. 1).

In this peripheral area, 12 growing sites of *C. foetida* were identified during field surveys. Under current climate conditions, the Maxent model with five climate variables (FFP, MAP, PAS, SHM, TAVE_SM) and two local-scale variables (SOIL and TWI; Fig. S5) was able to accurately explain the presence/absence of *C. foetida* (AUC = 0.958 on the train data, 0.965 on test data; Fig. S5). The response curves of the two local-scale variables (Fig. S5) confirmed that *C. foetida* largely prefers wet soils (high values of TWI) with slow mineralization of organic matter.

**Figure 2:**
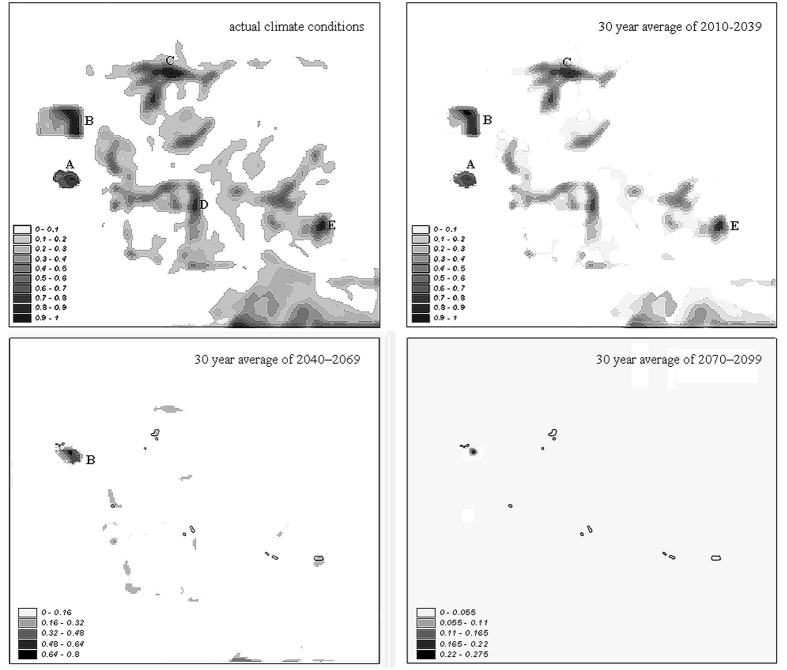
Suitability maps for translocation of *Carex foetida* under current (left; 1991–2009 climate period) and future climate conditions at the potential recipient site (i.e. peripheral area) for relocation (M. Cusna). Maxent suitability scores are indicated by different shades of grey. Letters A to E indicate the most suitable areas for species translocation. In the last two maps (bottom left and bottom right), polygons delineate the sites where the species is currently present. Maps were created using GRASS GIS^25^.

Under the current climate scenario, several parts of the peripheral area resulted in a suitability score >0.8 (Fig. 2). The locations where *C. foetida* is currently present received suitability scores ranging from 0.635 to 0.927. The minimum Maxent score (*S*_*M*_) of actual locations of *C. foetida* (i.e. 0.635) was used as minimum threshold of suitability for translocation. About 15.6 hectares of the study area were identified as suitable for *C. foetida* (*S*_*M*_ > 0.635). Five sub-areas (indicated with letters in Fig. 2), totalling about 10.6 hectares, were identified as particularly suited, as they also cover a large surface for translocation purposes.

The projections of the Maxent model to the three future climate scenarios (Table S2) are shown in Fig. 2. The short-term projection showed a suitability pattern similar to that of current climate conditions. Four sub-areas (about 9.1 hectares) out of five are expected to remain suitable (*S*_*M*_ > 0.635) for *C. foetida* in the 2010–2039 period. The medium-term projection showed a different suitability pattern, with only about 0.87 hectares of the peripheral area being suitable (*S*_*M*_ > 0.635) for *C. foetida* in the sub-area labelled B. The long-term climate projection showed no suitable areas, since the entire study area fell below the threshold value for *C. foetida*.

## Discussion

In our modelling framework for planning assisted colonization of climate-threatened plant species, we first defined an optimized climate profile of the study species in the core area, in the form of a reduced set of meaningful climatic variables. This step was performed in order to avoid statistical pitfalls as much as possible. In fact, as outlined by several authors, the full list of initial candidate variables may be oversized (one or more predictors may have little predictive power) and/or redundant (some predictors may be correlated in a significant manner, hence resulting in multicollinearity)^8^. This step also met the requirement for parsimony (i.e. with accuracy being approximately equal, the best model is the simplest one). Parsimonious models are more transferable to future conditions^9^,^10^. After the removal of unnecessary predictors, the Maxent AUC test helped validate the optimized set of climate variables.

In the second step, in order to preselect potential relocation sites, we applied a dissimilarity measure between the peripheral area and the mean vector of the optimized list of climate variables in the core area. The rationale behind this step is that excessive climate dissimilarities would *a priori* prevent any kind of assisted colonization. It followed the principle of cautious delimitation for the extent of out-of-range movements of organisms. In fact, accounting for possible differences in the ecological niche between current and recipient sites is necessary in determining whether assisted colonization is likely to incur high risks and should therefore be avoided and other types of conservation measures (seed banks and botanical gardens) promoted^6^.

In the third step, we calculated species suitability modelling in the peripheral area under current and future climate conditions, using the optimized set of climate variables defined in the first step with the addition of local-scale variables. This proved necessary because, when working on limited areas, local factors such as soil type and geomorphology could assume a non-negligible weight, besides climate factors^8^,^11^. As for climate variables, we avoided use of a comprehensive list of topographical variables and focused on two variables (SOIL and TWI) with known contributions to *C. foetida* potential distribution. The subsequent Maxent AUC test confirmed the fitness of our choice.

Maxent was appropriate for the proposed framework as it can deal with presence-only data and has better performance than other modelling algorithms^12^. Correlative species distribution models, such as Maxent, assume that species distributions are in equilibrium with the environment, which does not take into account the inability of individuals to reach a suitable habitat and may possibly lead to under-prediction of species current ranges^10^. In our case study this risk was absent, since the occurrence locations of *C. foetida* were monitored for many years both in the core area (1991–2014 period) and in the peripheral area (1999–2009). In case monitoring activities at the peripheral area are not feasible, we argue that the climate distance of the peripheral area with respect to the climate profile of the species at the core area may act as cost-effective and reasonable indicator to assess whether species distribution at the peripheral area is in equilibrium with its environment. For instance, in our case study climate similarity (i.e., Mahalanobis distance) indicated that around M. Cusna not any further area can provide a suitable habitat from a climatic viewpoint (Fig. 1), thus confirming the results achieved through field monitoring that the risk of under-prediction of species current ranges was absent.

For the purposes of our study, we opted to use the PRISM dataset instead of the Worldclim dataset^13^. This was mainly because the study species is climatically characterized by variables that are not present in the Worldclim dataset. In addition, the 1-km resolution of Worldclim climate data cannot capture fine-scale climate variability^14^.

Our results indicate that even the most promising site for translocation of *C. foetida* in the Emilia-Romagna region will be suitable only under current and short-term climate conditions. Thus translocation of *C. foetida* to the five sub-areas delineated here can be expected to be successful for about 25 years, up to the medium term (2040–2069). After this, the five sub-areas will most likely start to decline and are expected to become unsuitable by the beginning of the long-term period studied (2070–2099).

Results raise one main question: are assisted colonization activities worthwhile if they are only expected to be successful in the short or medium term? It is evident that a modelling approach like that proposed here can provide the basis to rigorously examine this question. It has been suggested that the success of assisted colonization activities also involves the population dispersing seeds into the surrounding countryside and producing satellite populations^15^. Our results indicated that not only is *C. foetida* likely to disappear from the peripheral area in the future due to climate scenarios, but also that in the N-Apennines mountain system (Emilia-Romagna region) there will be no further suitable sites for this species. In other words, producing satellite populations in surrounding areas is unlikely for *C. foetida* even under current climate conditions, and it will become increasingly improbable as time goes by.

The proposed approach (extra methodological details are presented in the Additional Supporting Information) deals with the spatio-temporal issues of species translocation. Further aspects should be considered when translocating plant species. Invasive alien species are a major threat to global biodiversity and ecosystem services^16^,^17^. Numerous plant species have been introduced in the past, and many have invaded large areas of natural vegetation and are still spreading. Some species change ecosystems, affecting their capacity to provide services such as water production, soil maintenance and nutrient cycling^18^. The proposed framework is not intended to replace decision-making tools for planning managing strategies to respond effectively to biological invasions. However, by considering the spatio-temporal feasibility of species translocations, it helps prevent the ill-advised introduction of many plant species as it limits the number of species and potential peripheral areas for which assisted colonization seems a suitable choice. Thus, it also circumscribes the introduction of potentially invasive alien species.

Moreover, we argue that the approach proposed here increases the practicality of the assisted colonization of plant species. In fact, the scale at which thousands of plant species would have to be moved to have any noticeable impact on mitigating climate change cannot be ignored. The costs for doing this work across the world are not negligible. Our approach, by circumscribing the reintroduction areas for which assisted colonization results suitable, provides researchers and conservation managers a tool to thoroughly limit their efforts to a restricted number of sites, and to identify species that will need other conservation actions in future when assisted colonization is not likely to be successful, such as storage in seed banks and/or botanical gardens.

We are aware that an eco-evolutionary response may reduce the risk of climate-driven extinction of some plant species. Even in absence of adaptation, phenotypic plasticity may partially counteract the negative effect of climate change. For instance, under moderate climate change, some snowbed species were recently observed to plastically respond to new environmental conditions^19^. Short and longer-term responses may also differ^20^,^21^,^22^, and extreme climatic events may cause complex responses of plant communities^23^. However we state that it seems logical and practical to assign higher priority to those plant species whose assisted colonization is more likely to be successful for the longest possible period as a result of the application of spatio-temporal modelling prior to *in situ* realization. For the remaining plant species, successive field studies might be realized in order to evaluate whether eco-evolutionary response and phenotypic plasticity can make assisted colonization useful despite the unfavorable projections.

Finally, by helping balance the risks and benefits of species translocation, the proposed approach has broad applicability as it can support the planning and assess the feasibility of constrained assisted colonization of any plant species in the face of climate change.

## Methods and Materials

### Species description, study areas and field sampling

The sedge *Carex foetida* All. (Cyperaceae) is a dominant species in igrophilous snowbeds of S-W European mountains characterized by high amounts of organic matter and nutrients^24^. The core area for *C. foetida* in Italy is in the Western Alps (Piemonte region; 25,388 km^2^; Fig. S1). Field surveys of *C. foetida* in this core area were conducted from 1991 to 2014. For each location detected, coordinates and height above sea level were recorded using a global positioning system (GPS). *C. foetida* is also currently present at the southern boundary of its distribution range in N-Apennines in the Emilia-Romagna region (22,184 km^2^; Northern Italy). Thus, our framework was applied to seek the most promising potential site for translocation (hereafter ‘peripheral area’) in the Emilia-Romagna region (Fig. S6) where our field sampling started in 1999 and finished in 2009, and the coordinates of each location were measured with GPS using differential correction techniques to improve the accuracy of data location (error <1 m). All maps were created using GRASS GIS^25^.

### Baseline climate data

To represent baseline climate conditions for both the core and the peripheral area, we used meteorological data for the period 1991–2009 calculated using the ClimateEU model^26^. It extracts and downscales PRISM (Parameter elevation Regressions on Independent Slopes Model) monthly data, and calculates seasonal and annual scale-free climate variables for specific locations based on latitude, longitude and elevation^27^.

We used the period 1991–2009 as the climate reference period, since it covered our field sampling period almost entirely. Eight biologically-relevant candidate climate variables that, to the best of our knowledge, are relevant to *C. foetida* physiological function and survival were selected (Table 1): annual heat:moisture index (AHM), degree-days below 0 °C (DD0), frost-free period (FFP), mean annual precipitation (MAP), mean annual temperature (MAT), annual precipitation as snow (PAS), summer heat:moisture index (SHM), summer (June-August) mean temperature (TAVE_SM).

### Climate profile of the study species in the core area

Maxent^28^ was used on the climatic dataset of the core area (Fig. S1) to optimize the set of candidate climate variables, i.e. to define a reduced set of meaningful predictor variables starting from the full list of candidate variables (Table 1). We employed the permutation importance test to assess the relative contribution of the climatic variables to the presence/absence of *C. foetida* in the core area. Values were normalized to give percentages^28^.

The Maxent model was calibrated using a random 70% of the data as a training sample, and evaluated using the remaining 30% as test data (split-sample approach)^29^. In order to minimize spatial autocorrelation^30^, we excluded one sampling point for each pair of species locations that resulted in a distance less than 100 m. We selected the logistic model output that represents a probability estimate of habitat suitability for each pixel. To evaluate the predictive accuracy of the optimized set of climate predictors, we used the Receiver Operating Characteristic (ROC) curve with AUC score on both training and test data.

### Climate distance of the peripheral area

In order to select the most promising site for translocation of *C. foetida* in the Emilia-Romagna region, we applied a dissimilarity measure between the climate profile of the study species in the core area (see previous section) and the potential peripheral areas.

Mahalanobis distance (*D*_*M*_) is a widely-used method for measuring how similar/dissimilar a set of conditions is to a reference set^31^. We computed *D*_*M*_ for the whole mountain system (areas >800 m a.s.l.; 3520 km^2^) of the Emilia-Romagna region. To do this, we used the ClimateEU model to create the raster layers (cell size = 0.1 Km^2^) of the climate variables for the Emilia-Romagna region using the 1991–2009 climate period to represent the current climate conditions (Fig. S2).

### Current potential distribution of the study species in the peripheral area

To represent current climate conditions in the peripheral area, we again employed the 1991–2009 climate period using the ClimateEU model for the optimized set of climate variables (climate profile of the study species). Besides the climate predictors, for the peripheral areas we added two local-scale predictors (Table 1) with known influence on the study species: soil type (SOIL, i.e. mineralization rate) and accumulation of overland water flow (TWI, topographic wetness index). In fact, *C. foetida* prefers wet soils with slow mineralization of organic matter^24^.

### Future potential distribution of the study species in the peripheral area

To represent future climates in the peripheral area, we used projections of the CMIP5 multimodel data set^32^. In order to limit the modelling effort, we worked with an ensemble mean of all available model runs for the A2 emission scenario^33^. We excluded poorly validated AOGCMs (MIROC3.2, MRI-CGCM2.3.2, MIROC3.2, IPSL-CM4, FGO-ALS-g1.0, GISS-ER, GISS-EH, and GISS-AOM)^33^. Three future periods were studied, here referred to as short term (30-year average of 2010–2039), medium term (30-year average of 2040–2069) and long term (30-year average of 2070–2099)^34^. The Maxent model calibrated on current climate data was applied to the three future periods. The resulting potential distributions of *C. foetida* under current and future climate conditions were cartographically explored to identify sub-areas suitable for *C. foetida* for the longest possible period.

## Additional Information

**How to cite this article**: Ferrarini, A. *et al*. Planning for assisted colonization of plants in a warming world. *Sci. Rep.* **6**, 28542; doi: 10.1038/srep28542 (2016).

## Supplementary Material

Supplementary Information

## Figures and Tables

**Table 1:** List of the candidate variables tested as eligible predictors of presence of *Carex foetida* in the core area (Piemonte region, Northern Italy) and in the potential site (i.e. peripheral area) for species translocation.

Candidate variable	Code	Data type	Units	Used for area (*)
Annual heat:moisture index	AHM	Continuous	°C mm^−1^	Core and peripheral
Degree-days below 0 °C	DD0	Continuous	°C	Core and peripheral
Frost-free period	FFP	Continuous	unitless	Core and peripheral
Mean annual precipitation	MAP	Continuous	mm	Core and peripheral
Mean annual temperature	MAT	Continuous	°C	Core and peripheral
Precipitation as snow	PAS	Continuous	mm	Core and peripheral
Summer heat:moisture index	SHM	Continuous	°C mm^−1^	Core and peripheral
Summer (Jun.-Aug.) mean T°	TAVE_SM	Continuous	°C	Core and peripheral
Soil type	SOIL	Categorical	2 categories	Peripheral
Topographic wetness index	TWI	Continuous	m^2^ m^−1^	Peripheral

*Depending on the analysis of variable contributions (only if permutation importance was >1).
