# Supplementary material for: Planning for assisted colonization of plants in a warming world
Source: Sci Rep. 2016 Jun 27;6:28542. doi: 10.1038/srep28542 (PMC4921867; doi:10.1038/srep28542)
Supplement: Supplementary Information [file srep28542-s1.pdf]

Supplementary material for "Planning for assisted colonization of plants in a warming world"

Alessandro Ferrarini[1], Alberto Selvaggi[2], Thomas Abeli[1], Juha M. Alatalo*[3], Simone Orsenigo[4], Rodolfo Gentili[5], Graziano Rossi[1]

[1] Department of Earth and Environmental Sciences, University of Pavia, via S. Epifanio 14, I-27100 Pavia, Italy

[2] I.P.L.A., Corso Casale 476, I-10132 Torino, Italy

[3] Department of Biological and Environmental Sciences, College of Arts and Sciences, Qatar University, P.O. Box 2713, Doha, Qatar

[4] Department of Agricultural and Environmental Sciences - Production, Landscape, Agroenergy, University of Milan, Via Celoria 2, I-20122 Milano, Italy

[5] Department of Environmental and Territory Science, University of Milano-Bicocca, Piazza della Scienza 1, I-20126 Milano, Italy

* Corresponding author

E-mail: jalatalo@qu.edu.qa

Supplementary figures

Fig. S1…………………………………………………………………………………..2

Fig. S2…………………………………………………………………………………..3

Fig. S3…………………………………………………………………………………..4

Fig. S4…………………………………………………………………………………..5

Fig. S5…………………………………………………………………………………..6

Fig. S6…………………………………………………………………………………..7

Supplementary tables

Table S1 ……………………………………………………………………………..8

Table S2 ……………………………………………………………………………..9

Additional Text

Extra methodological details……………………………………………..…………………10

Fig. S1: Core area for *C. foetida* in Italy (Piemonte region; Northern Italy; 25,388 km^2). To represent current climate conditions of *Carex foetida* (red points; field sampling period: 1999-2009), we used meteorological data for the period 1991-2009 using the ClimateEU model. Eight biologically-relevant candidate climate variables were assigned to each location of *C. foetida*. These were: annual heat:moisture index (AHM), degree-days below 0°C (DD0), frost-free period (FFP), mean annual precipitation (MAP), mean annual temperature (MAT), annual precipitation as snow (PAS), summer heat:moisture index (SHM), summer (June-August) mean temperature (TAVE_SM). Map was created using GRASS GIS [1].

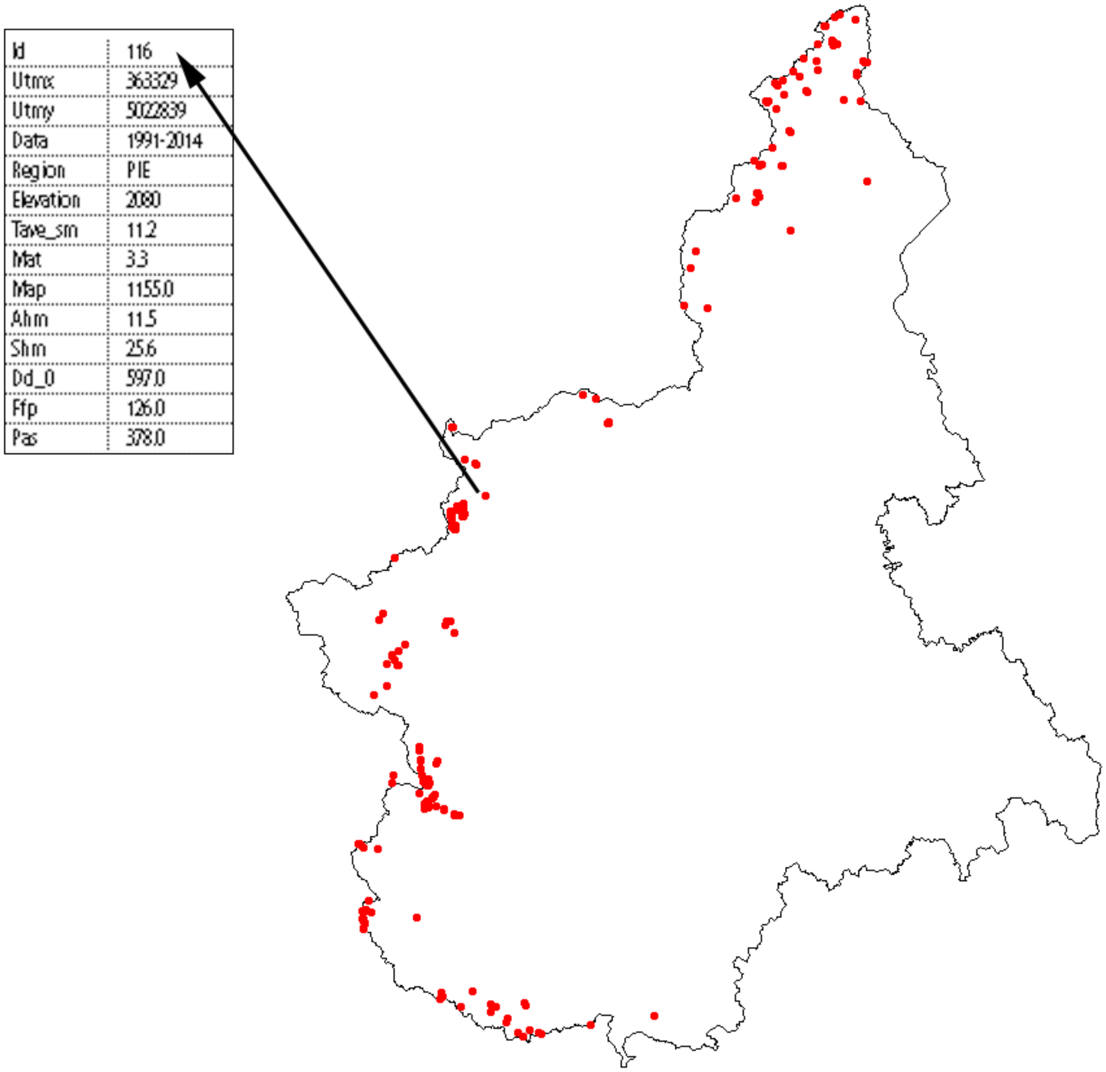

Fig. S2: Results of the Maxent suitability model for *Carex foetida* in the core area (Piemonte region) under current climate conditions. Left: Accuracy assessment through Receiver Operating Characteristic (ROC) and Area Under the Curve (AUC) analyses. Right: Response curves of the optimized set of climate predictors (FFP: Frost-free period; MAP: Mean annual precipitation; PAS: Precipitation as snow; SHM: Summer heat:moisture index; TAVE_SM: Summer (Jun.-Aug.) mean T°).

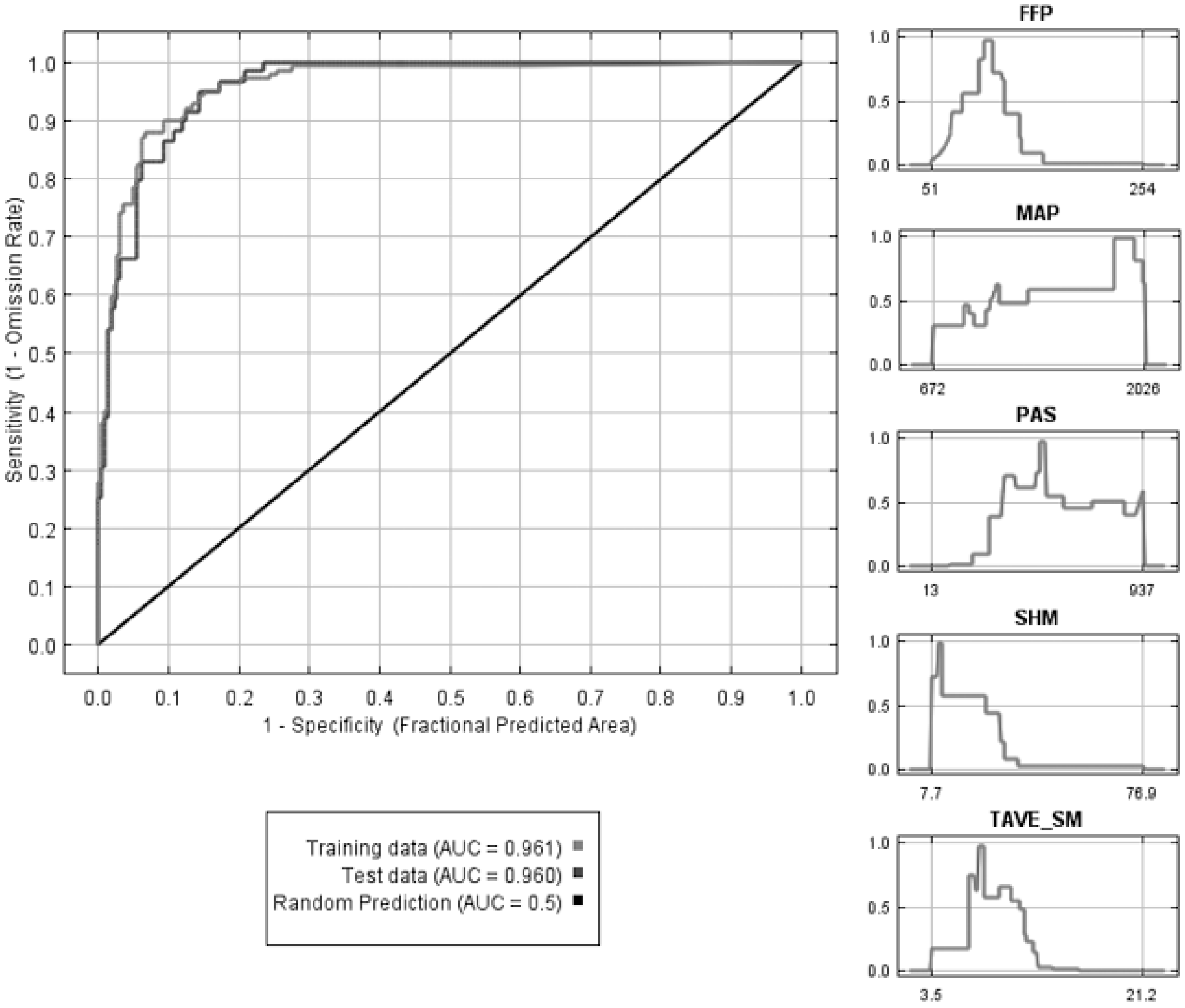

Fig. S3: The area around M. Cusna resulted the most promising area for translocation of *Carex foetida* in the Emilia-Romagna region, Northern Italy. This area extends over 575 ha (about 2500 m x 2300 m; barycentre coordinates: 44°15'N, 10°24'E), at altitude 1370-2120 m a.s.l.. The site is within an EU Site of Community Importance (SCI IT4030004 Val d'Ozola-Monte Cusna) under the European Habitat Directive (92/43/EEC). Map was created using GRASS GIS [1].

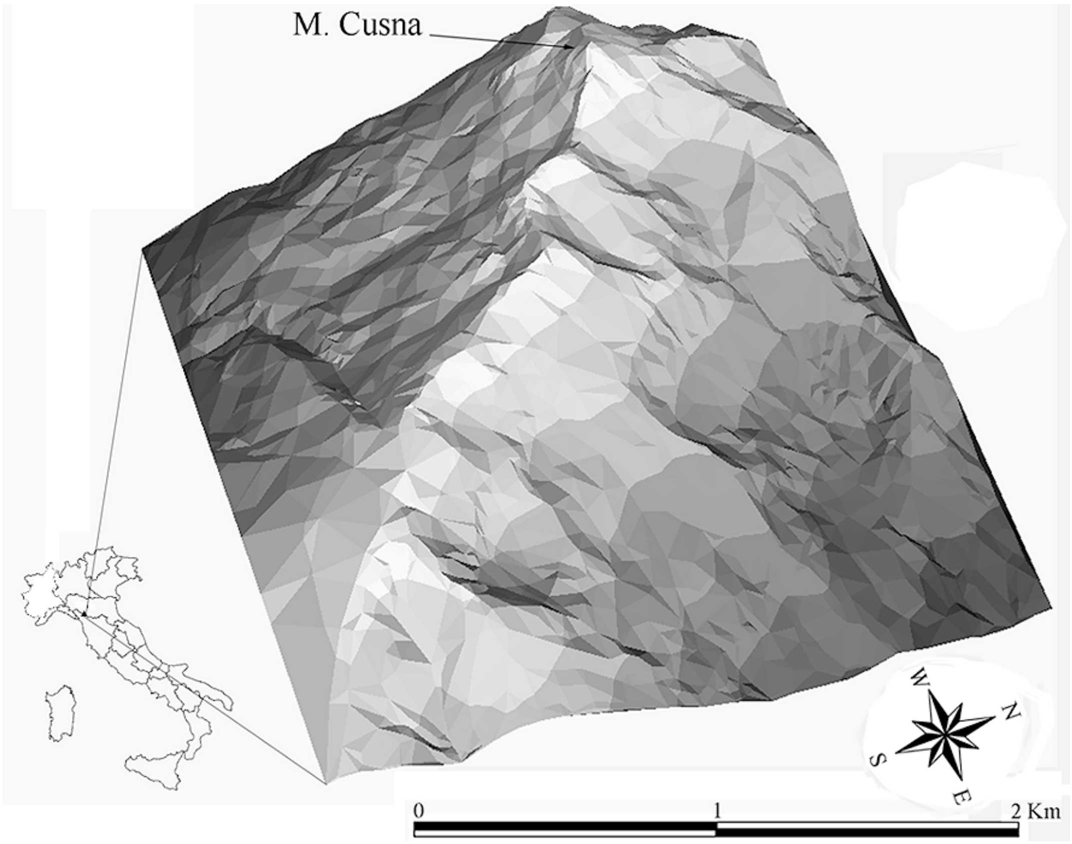

Fig. S4: Climate similarity (measured as Mahalanobis distance) of the peripheral area (M. Cusna; about 2500 m x 2300 m) with respect to the climate profile of *Carex foetida* in the core area (Piemonte region, Northern Italy). Lower values (i.e. lower distances) indicate higher similarity with respect to the climate conditions of *C. foetida* in the core area, higher values (i.e. higher distances) stand for lower similarity. Map was created using GRASS GIS [1].

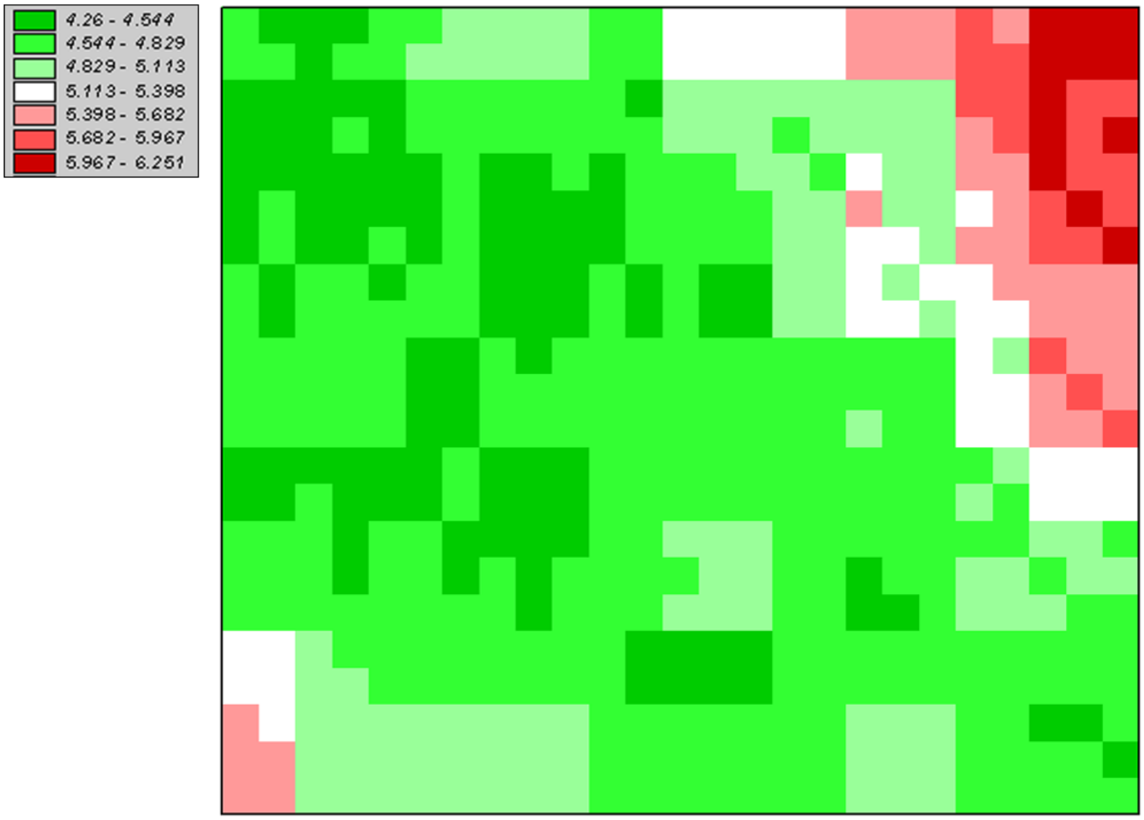

Fig. S5: Results of the Maxent suitability model for *Carex foetida* at the potential recipient site for relocation (M. Cusna) under current climate conditions (1991-2009 climate period). Left: Accuracy assessment through Receiver Operating Characteristic (ROC) and Area Under the Curve (AUC) analyses. Right: Response curves of the two local-scale predictors, SOIL (1 = fast mineralization of organic matter; 2 = slow mineralization of organic matter) and topographic wetness index (TWI) used along with the optimized set of climate variables.

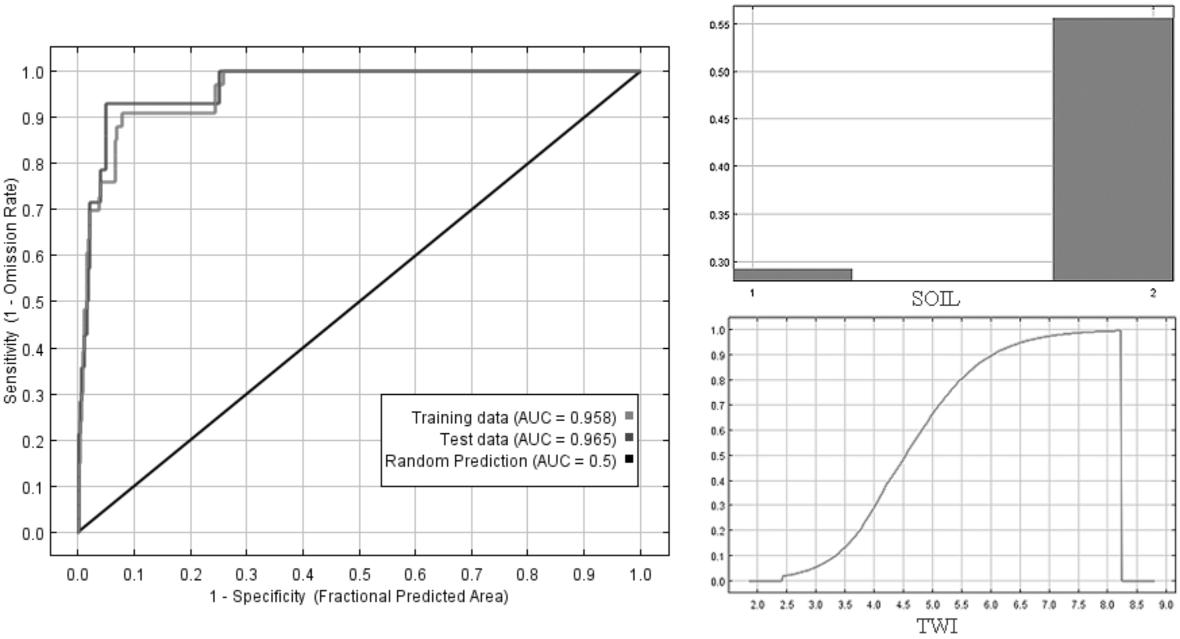

Fig. S6: Emilia-Romagna region (Northern Italy; 22,184 km^2). To represent current climate conditions, we used meteorological data for the period 1991-2009 using the ClimateEU model. To do this, we created the raster layers (cell size = 1 ha) of the climate variables for the whole mountain system of the Emilia-Romagna region. Eight biologically-relevant candidate climate variables were assigned to each cell. These were: annual heat:moisture index (AHM), degree-days below 0°C (DD0), frost-free period (FFP), mean annual precipitation (MAP), mean annual temperature (MAT), annual precipitation as snow (PAS), summer heat:moisture index (SHM), summer (June-August) mean temperature (TAVE_SM). Map was created using GRASS GIS [1].

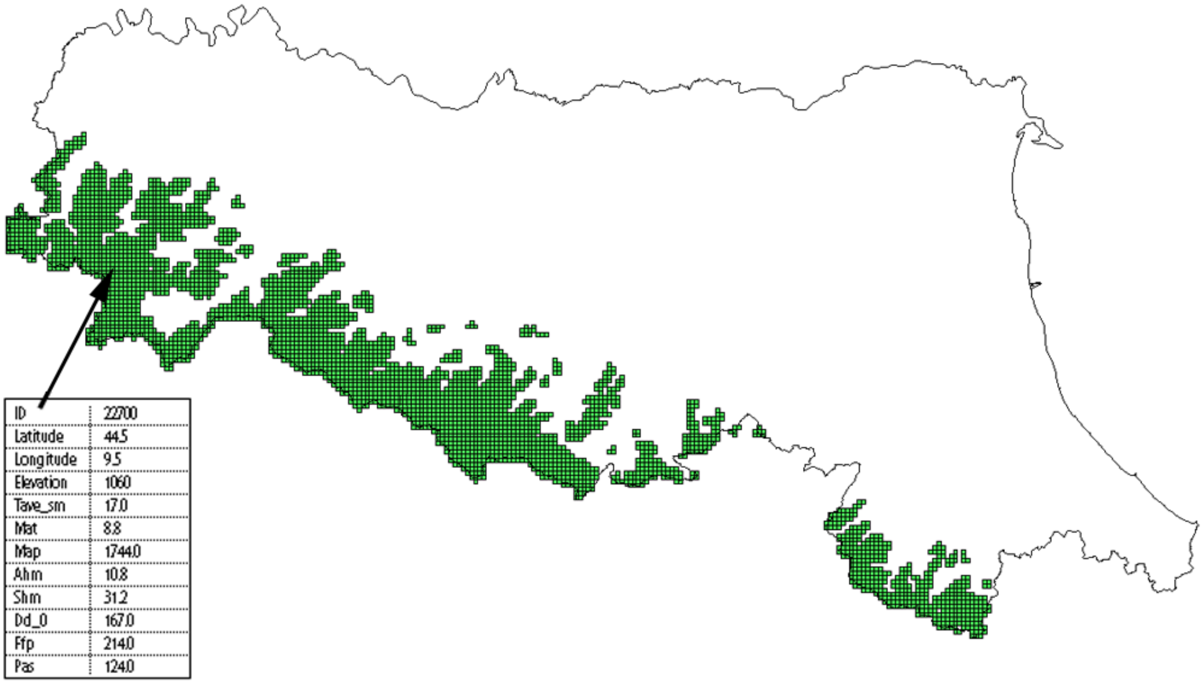

Table S1: The resulting climate profile (202 sampling points; climate period 1991-2009) of the study species *Carex foetida* in the core area (Piemonte region).

**Variable**	**Mean**	**Std. dev.**	**Minimum**	**First quartile**	**Median**	**Third quartile**	**Maximum**	**Permutation importance**
FFP	107.9	19.26	68	93	108.5	120	160	2
MAP	1206	288.2	807	1025.7	1092.5	1351	2129	16.7
PAS	517.5	175.2	169	386.8	494	608.5	1223	9.1
SHM	20.77	6.17	5.6	17.02	21	25.1	39.8	54.1
TAVE_SM	9.04	1.61	4.4	7.8	9.15	10.1	13.1	18.1

Table S2: Average climate changes expected at the potential site for species translocation (M. Cusna; *n*= 550 pixels) for the five climate variables selected to profile *Carex foetida*.

	**Baseline (1991-2009)**	**Period 2010-2039**	**% change compared with baseline**	**Period 2040-2079**	**% change compared with baseline**	**Period 2080-2099**	**% change compared with baseline**
FFP	169.9	175.2	+3.12	187.9	+10.59	208.1	+22.48
MAP	1528.8	1857.0	+21.47	1783.5	+16.66	1695.8	+10.92
PAS	401.7	476.2	+18.55	356.9	-11.15	235.2	-41.45
SHM	27.0	25.6	-5.19	31.0	+14.81	42.0	+55.56
TAVE_SM	13.0	13.2	+1.54	14.7	+13.08	16.7	+28.46

Extra methodological details

Mahalanobis distance

Mahalanobis distance (D_M) [2], was calculated as:

$$D_M(\vec{x},\vec{m}) = \sqrt{(\vec{x}-\vec{m})^T C^{-1}(\vec{x}-\vec{m})} \qquad (1)$$

In our case study, $\vec{x}$ represents the vector of climate data in the peripheral area (i.e. the generic i^{th} pixel is represented by its vector $\vec{x}_i$ of climate data), $\vec{m}$ is the vector of the average climate variables in the core area, C^{-1} is the inverse covariance matrix of the climate variables in the core area and T indicates vector transpose. This unitless distance is zero if $\vec{x}$ is equal to $\vec{m}$ and grows as $\vec{x}$ moves away from $\vec{m}$.

Local scale variables

Besides the climatic variables, a digital elevation model (DEM) of the peripheral area was obtained by digitizing available topographical maps of the Emilia-Romagna Region. The DEM at 1:10,000 scale was then used to derive the wetness index. Topographical wetness index (TWI hereafter) estimates the accumulation of overland water flow and it is used in vegetation studies to represent the spatial variability of soil wetness [3]. It was calculated as:

$$TWI_i = \ln\left(\frac{c_i}{\tan s_i}\right) \qquad (2)$$

where TWI_i is the wetness index at location i, c_i is the catchment area (the area draining into a pixel, expressed in $m^2\ m^{-1}$) and s_i is the slope angle (in degrees) at location i [4].

A soil map of the study area at 1:10,000 scale was provided by the Emilia-Romagna regional council. It was reclassified into two categories: soils with a) fast and b) slow mineralization of organic matter. In the study area, the former category corresponds to decarbonated soils with strong profile differentiation and frequent weak acidification in surface horizons (Eutric Cambisols)

and soils with weak profile differentiation (Eutric Leptosols or Eutric Regosols). The latter category corresponds to strongly or very strongly acidified soils with weak (Umbric Leptosols) or strong (Humic Cambisols, Haplic Podzols) profile differentiation.

The resulting set of predictor variables (optimized set of climate variables + local-scale predictors) was used to assess species potential distribution modelling under current climate conditions (1991-2009 period) using the Maxent methodology. All maps were created using GRASS GIS [1].

Future potential distribution in the peripheral area

The Maxent score (S_{Mi}) of the generic i^{th} pixel of the peripheral area, over short, medium and long periods, changed with variation in the climate vector $\vec{x}_i$ at that pixel, while the local-scale variables ($SOIL_i$ and TWI_i) at that pixel were kept constant:

$$\Delta S_{Mi} = f(SOIL_i,\ TWI_i,\ \Delta\vec{x}_i) \quad (3)$$

References

1. GRASS Development Team. *GRASS GIS 7.0*. Fondazione E. Mach - Research and Innovation Centre, S. Michele all'Adige, Italy (2015). URL: https://grass.osgeo.org/
2. Mahalanobis, P. C. On the generalized distance in statistics. *Proc. Natl. Inst. Sci. Calcutta* **2,** 49–55 (1936).
3. Parolo, G., Rossi, G. & Ferrarini, A. Toward improved species niche modelling: Arnica montana in the Alps as a case study. *J. Appl. Ecol.* **45,** 1410–1418 (2008).
4. Moore, I. D., Grayson, R. B. & Ladson, A. R. Digital terrain modelling: a review of hydrological, geomorphological, and biological applications. *Hydrol. Process.* **5,** 3–30 (1991).
